# Advancements in Algal Microbiome Research: A Game-Changer for Climate Resilience and Invasion Success?

**DOI:** 10.1007/s00248-025-02563-8

**Published:** 2025-06-10

**Authors:** María Vila Duplá

**Affiliations:** Institute of Water Research, University of Granada, Ramón y Cajal, 4, 18071 Granada, Spain

**Keywords:** Microbiome, Macroalgae, Dysbiosis, Climate change, Global stressors, Invasive species

## Abstract

While marine microbiomes have been getting more attention in recent years, they remain understudied compared to those of terrestrial systems. With the refinement of molecular methods, microbiome research has extended to other key marine organisms such as macroalgae. The microbiome plays a key role in macroalgal health, adaptation to environmental conditions, and resilience to climate stressors. The main factors affecting the algal microbiome are host specificity (genetics, functional profile, phylum and species identity), life stage, morphology, thallus region, and tissue age. Other significant drivers of microbiome community structure include spatiotemporal distribution and environmental conditions, especially as global stressors intensify with climate change. The mechanisms through which the microbiome of invasive seaweeds might enhance their competitiveness over native species are still unclear. However, there is evidence that, like climate resilience, invasive potential is linked to the functional flexibility of associated microbiota, allowing the host to adapt to the new environmental conditions. The main objective of this review was to synthesize the current understanding of the macroalgal microbiome and propose future directions in microbiome research based on identified shortcomings. Based on the knowledge gaps detected, there is an urgent need for multi-factorial experimental studies that link host and microbiome gene expression through chemical signals under future climate change scenarios, standardization of analytical methods, and a focus on underrepresented geographical regions and species. While algal microbiome research holds great promise for predicting and mitigating the effects of climate change and invasive species, embracing new tools and tackling ecologically relevant mechanistic and applied questions will be essential to advancing this field.

## Introduction

A microbiome is a specific microbial community occupying a well-defined habitat or host, with distinct physicochemical properties and functions, and its interactions with the surrounding environment [1]. All the living microorganisms that are part of a microbiome (i.e., bacteria, archaea, algae, and fungi) are referred to as microbiota [2]. By these definitions, non-living microbial components (e.g., proteins, lipids, polysaccharides, and nucleic acids) and other structural elements (microbial metabolites and environmental conditions), together with the microbiota, form the microbiome. Microbiota are essential to the algal host as they play crucial growth-promoting and protective roles, forming a tight relationship that is analogous to the holobiont concept in coral ecology [3].

Although the components of the microbiome are constantly changing, it is useful to identify core microbiota (i.e., permanent members of the microbiome for a given host genotype or environment, often carrying out essential functions for the host) [4] and keystone taxa (i.e., members of the microbiota that have a major influence on microbiome composition and function regardless of their abundance) [5]. These key players contribute to nutrient cycling, disease suppression, and ecological interactions, and their functions are increasingly recognized as vital for the health and stability of ecosystems [3]. Interestingly, rare taxa also play an essential role across global microbiome communities through the production of antimicrobial toxins that regulate the growth of dominant taxa and enhance biodiversity [6]. Due to the high diversity of associated microbiota in some host organisms, defining their microbial components using molecular techniques can be very challenging [7].

The close link between hosts and their associated microbiota is likely the product of coevolution, and in order to have a comprehensive understanding of the processes affecting either one, they need to be studied conjointly [8]. Host and microbiome exert a mutual influence on one another, which makes discerning the direct versus indirect effects of external drivers extremely challenging [9]. On the one hand, a host may modulate its microbiome to dynamically adjust to changes in the environment [10], and biological and ecological traits of the host such as population density [11] and morphological complexity [12] directly affect microbiome diversity. On the other hand, microbiome genetic and functional composition influence the host’s health, ecological interactions, and resilience to external stressors [13]. In fact, disease development and worsening in host organisms, particularly in marine ecosystems, is often attributed to dysbiosis [14]. Dysbiosis refers to a disruption of the microbiome leading to imbalances in the microbiota and shifts in their functional composition and metabolic activity that can cause diseases in the host [15]. Interestingly, microbiome community composition is more variable among stressed hosts than healthy ones [16], possibly because stressors reduce the host’s ability to regulate microbiome composition.

The main objective of this review is to synthesize the current understanding of the macroalgal microbiome and propose future directions in microbiome research based on identified shortcomings. This study aims to answer the following questions: (i) What are the current knowledge gaps and limitations in the study of the algal microbiome? (ii) How does it contribute to its host’s health, and to its resilience in the face of environmental changes? (iii) What factors regulate the algal microbiome and mediate host-microbiome interactions? (iv) Does it mitigate or exacerbate the effects of climate change? and (v) How and to what extent does it influence the invasion success of non-native macroalgae? By establishing a clearer framework for understanding these complex relationships, this review intends to provide direction for future research and inform practical applications in marine ecosystem management.

## Research Trends and Knowledge Gaps in Microbiome Ecology

While marine microbiomes have been getting more attention in recent years, and particularly during the last decade, they remain understudied compared to those of terrestrial systems. Research on marine microbiomes has focused largely on animal hosts, particularly sponges [3] and corals [17], as these systems are highly impacted by climate change, disease, and other anthropogenic pressures [18]. With the refinement of methods for the study of microbiomes in the past few years, microbiome research has extended to other marine organisms such as macroalgae. In a recent study, 14 microbiome core genera were identified across the three macroalgal phyla, accounting on average for over half of the bacterial abundances, and with genomic potential to degrade algal polysaccharides and produce bioactive secondary metabolites [19]. Despite their ecological importance, kelp microbiomes remain relatively underexplored compared to their coral counterparts, with most studies focusing on the role of epiphytic bacteria [19, 20], while other key microbial groups such as fungi and microalgae receive less attention.

To identify knowledge gaps in marine microbiome research, systematic literature searches were conducted using the Scopus database on February 22nd, 2025. In addition to its comprehensive coverage of marine and environmental science research, Scopus was selected due to its higher number of indexed journals and lower provision of irrelevant searches compared to other commonly used databases [21, 22]. Boolean operators were applied to construct search queries that combined relevant scientific and common terms (e.g., “macroalgae” OR “seaweeds”) through sequential inclusion (e.g., “microbiome” AND “macroalgae”). The search was restricted to research articles published in English and containing the selected terms within the title, abstract, or keywords. Results were screened manually and further filtered and organized by country of publication and year to analyze temporal and geographic trends in the literature.

**Fig. 1:**
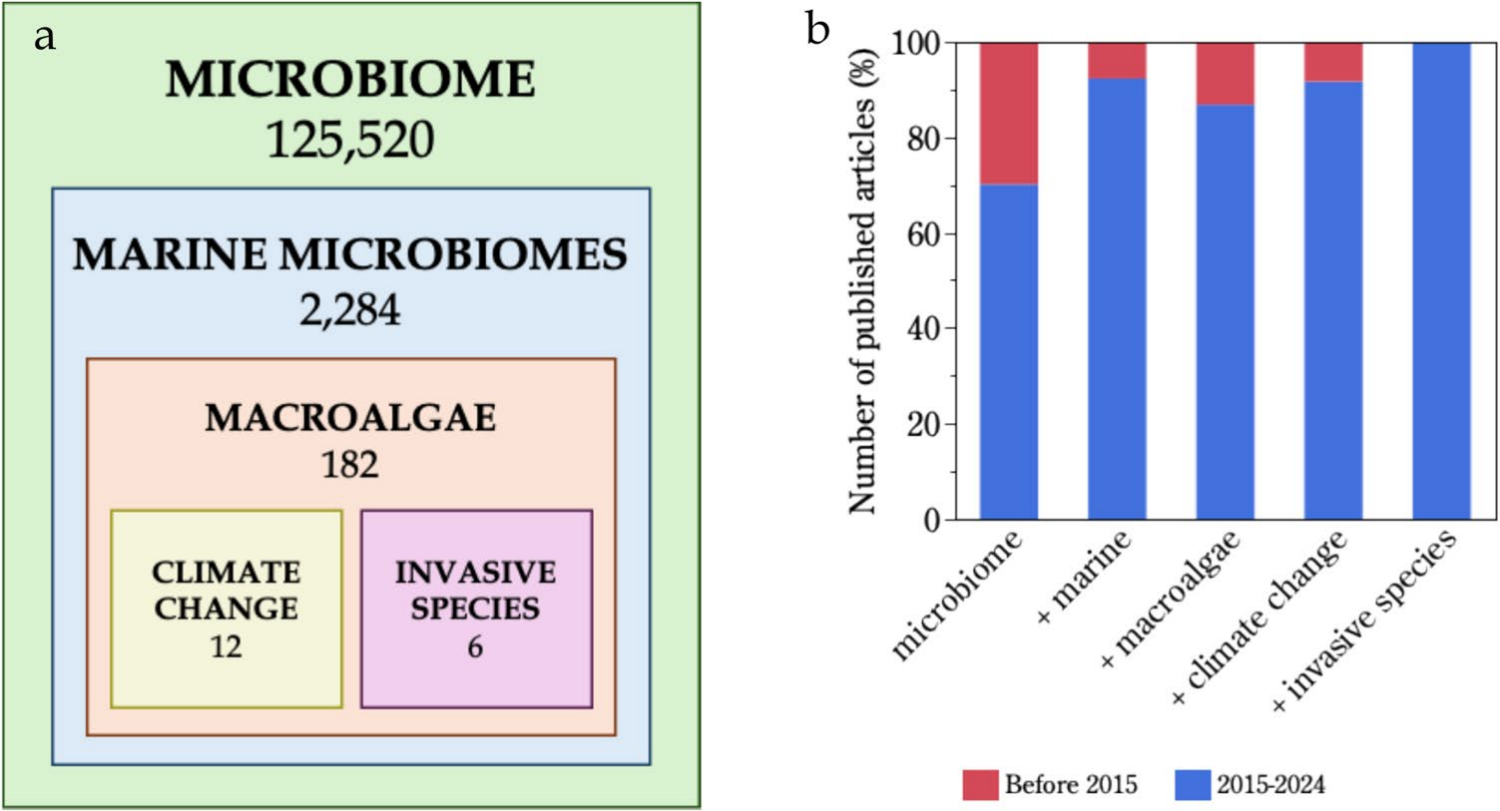
Results of systematic literature searches in Scopus including (**a**) the total number of published scientific articles focusing on microbiome research and containing each of the key terms (sequentially inclusive) in their title, abstract, or keywords, and (**b**) the percentage of articles published before or within the last decade including these key terms

The results of these systematic searches bring attention to critical knowledge gaps in marine microbiome research, with articles focusing on marine microbiome representing less than 2% of the total microbiome research. Moreover, only 8% of these articles studied the macroalgal microbiome, and only 7% of these investigated the microbiome of macroalgae in the context of climate change. More importantly, the potential role of algal microbiomes in mediating the invasive capacity of non-native species, a rising global issue, has been largely overlooked (Fig. 1a). This analysis also highlights microbiome ecology as an emerging research area, as evidenced by the growing body of scientific literature in recent years. Specifically, 70% of research articles about microbiomes, and over 90% of the articles on marine microbiomes, were published within the last decade. This was also the case for over 90% of articles relating macroalgal microbiomes to climate change, and 100% of the articles linking macroalgal microbiomes to invasive success (Fig. 1b).

## The Macroalgal Microbiome: An Understudied Ecosystem

Compared to more extensively studied microbiomes, including those of corals, sponges, and terrestrial plants, the macroalgal microbiome remains significantly underrepresented in the literature. In coral holobionts, the microbial diversity and functional interactions have been well characterized, with studies highlighting the roles of bacteria, archaea, viruses, and eukaryotic microorganisms in coral health and resilience [23, 24]. Similarly, over 40 bacterial phyla, which contribute to nutrient cycling and chemical defense mechanisms, have been identified in the highly diverse microbiota of sponges [25, 26]. In terrestrial plants, microbiomes research has revealed consistent core microbiota that play crucial roles in nutrient uptake, stress tolerance, and disease resistance [27, 28]. In contrast, studies on macroalgal microbiomes are fewer and often limited in scope, highlighting a significant gap in our understanding of marine holobionts and emphasizing the need for more comprehensive and systematic research in this area [29].

**Fig. 2:**
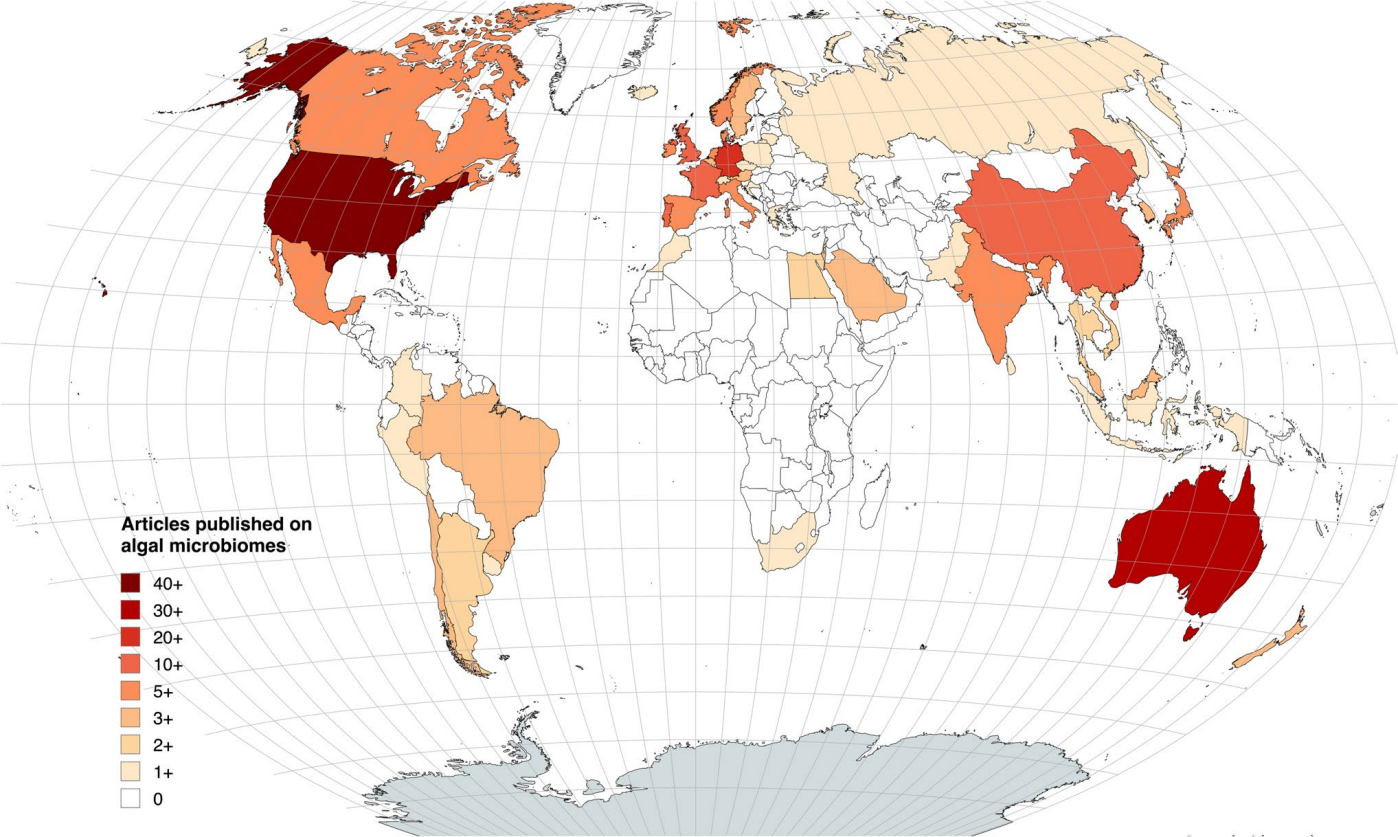
Map representing the scientific production (total number of articles published) on algal microbiomes by country. Data are extracted from systematic literature searches in Scopus

The microbiome of macroalgae is highly dynamic, influenced by factors such as host species, environmental conditions, and interactions with other organisms [30]. Microbiome biodiversity of macroalgae is often underestimated, with most studies focusing exclusively on the bacterial community [31]. Other members of the microbiome that are also essential in shaping its overall structure and function, such as microalgae, fungi, archaea, and viruses, have received less attention [3]. Moreover, until recently, microbiome studies had primarily focused on taxonomic diversity, with only a few studies investigating the functional potential of microbiota [9, 32]. Consequently, there is still a need to build a comprehensive framework for assessing the functional roles of microbiota across different macroalgal species and ecosystems. Unfortunately, there is a clear under-representation of certain geographical areas in algal microbiome research, particularly from underdeveloped regions (Fig. 2). The bias towards marine ecosystems in the Northern Hemisphere is concerning, as it overlooks critical habitats in continents like Africa, with available data failing to represent the full microbiome diversity of global ecosystems.

Although host specificity is one of the main factors influencing microbiota [33], the great majority of studies are limited to the study of “model” kelp species such as *Macrocystis pyrifera* (giant kelp) and *Nereocystis leutkeana* (bull kelp), and other commercially valuable species [31, 34, 35]. Chlorophytes are the most unrepresented phylum in algal microbiome research, followed by rhodophytes [29]. With increasing environmental stress on macroalgae, there is an urgent need for studying the microbial taxa across all life history stages of host organisms, as this remains a critical research gap [36]. Another key limitation in current research is the lack of longitudinal studies that track stressor-induced microbiome changes over time. The majority of studies provide a snapshot of microbiome diversity at a single point in time, which limits our understanding of microbiome’s short- and long-term responses to environmental change [37]. Furthermore, the lack of standardization in sampling methods, especially in underrepresented areas like the Southern Hemisphere, skews our understanding of global marine microbiomes [38].

Recent technological and methodological advances have allowed for more precise characterization of macroalgal microbiomes, though there is still no standardized protocol in microbiome research. A widely used approach to quantify microbiota in macroalgae is 16S rDNA amplicon sequencing [39]. Despite advancements including multi-omics, improved extraction methods, and development of specialized primers [40, 41], quantifying the core microbiome taxa remains challenging due to inconsistencies in analytical protocols [42], which introduce variability in the data [7]. This lack of standardization is further complicated by the high variability in macroalgal host species, including differences in tissue structure, surface chemistry, and production of antimicrobial compounds, all of which influence microbial colonization and persistence. For instance, macroalgae produce a variety of antimicrobial compounds that can affect the composition of their associated microbiota [43].

Additionally, taxonomic classifications of microbial molecular data heavily rely on the availability of comprehensive and high-quality reference databases. However, there remains a significant gap in genomic representation of marine microbes, especially compared to those from terrestrial and freshwater environments. This limitation reduces the accuracy of taxonomic assignments and constrains functional interpretation of microbial community data [44]. While technological improvements continue to advance the field, challenges related to contamination, database completeness, and host-specific variability remain critical barriers to studying macroalgal microbiomes effectively.

## Friend or Foe? How the Microbiome Influences the Health of Its Algal Host

**Fig. 3:**
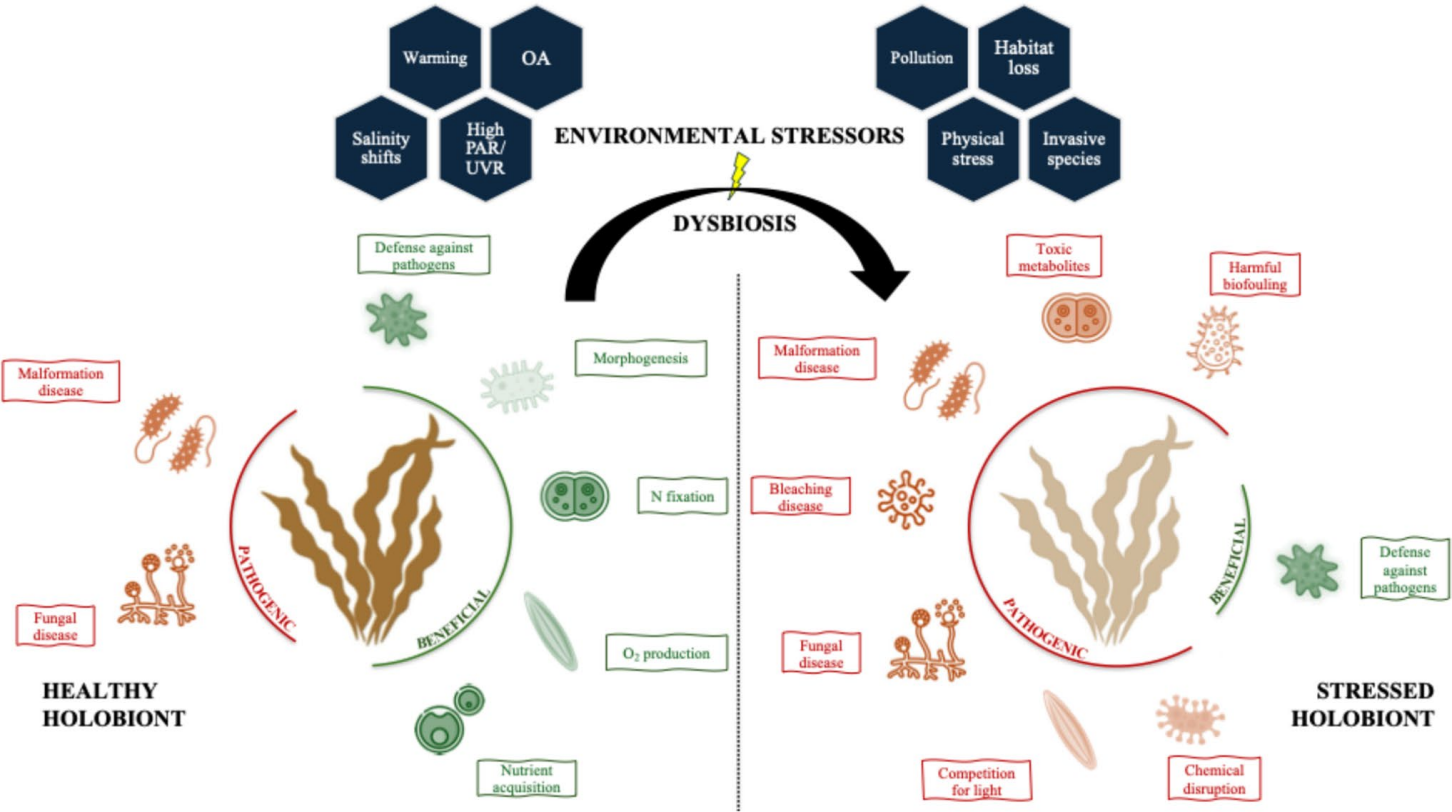
Impact of environmental stressors (climatic, natural, and anthropogenic) on the macroalgal microbiome, including composition and functional changes linked to dysbiosis. Under environmental stress, benign microbiota may become pathogenic, putative pathogens increase, and key functions are lost, often leading to disease. Microbiome diversity is overall higher in stressed than healthy holobionts, as new microbial taxa and functions emerge to adapt to the new environmental conditions

The macroalgal microbiome plays a critical role in host health, encompassing a spectrum of interactions, from beneficial symbioses that enhance growth and defense to pathogenic associations that can lead to disease and tissue degradation [3]. Understanding these dynamics is crucial, as environmental stressors can shift the balance of microbiota, influencing the overall health and resilience of macroalgal hosts (Fig. 3).

### Beneficial Interactions

The macroalgal microbiome can have a symbiotic relationship with its host, promoting its growth [3, 45], morphogenesis [46], and defense [47, 48] under non-stressful environmental conditions across different taxa. In fact, bacteria isolated from algal microbiomes only express inhibiting properties against alien microorganisms in the presence of the algal host [49], indicating adaptation to an epiphytic lifestyle [50]. Microbiome community abundance directly affects propagule success in *M. pyrifera*, modulating its recruitment success and the health of kelp forest ecosystems [51]. Kelp-specific microbiome taxa often include bacteria that prevent the colonization of eukaryotic larvae and exhibit antibacterial activity [52]. Similarly, Ghaderiardakani et al. [53] found that morphogenesis and resilience of Antarctic *Ulva* spp. depend on algal growth and morphogenesis-promoting factors released by cold-adapted bacteria. The presence of certain bacterial groups with specific ecological strategies within the microbiome is linked to harmful macroalgal blooms such as the green tides caused by *Ulva prolifera* [54]. Among the most common growth-promoting bacteria are *Maribacter* sp. and *Sulfitobacter* sp., both found in the microbiome of red and green algae [55]. In many brown algae, the predominant family are Proteobacteria, which produce organic compounds called siderophores that can enhance algal growth under iron-deficient conditions [50].

Epiphytic microorganisms can increase nutrient availability to their macroalgal hosts by converting nutrients into bioavailable forms, as observed in *Nereocystis leutkeana* [56]. Beyond inorganic nutrients, microbial symbionts may also contribute essential organic compounds. For instance, several macroalgal-associated bacteria possess genes for synthesizing vitamins such as B_12_, which are crucial for algal metabolism but cannot be synthesized by the hosts themselves [39, 57]. This aligns with broader findings across algal systems demonstrating widespread auxotrophy (i.e., the inability to synthesize a particular organic compound needed specifically for growth) for B_12_ and other vitamins, as shown in the model green alga *Chlamydomonas reinhardtii* [58]. Metabolic exchange has been directly shown in co-culture experiments, which found reciprocal metabolite transfer between *Lobomonas rostrata* and vitamin B_12_-producing bacteria [59]. Similarly, in *Saccharina japonica*, folate biosynthesis genes expressed by associated bacteria have been linked to microbial community stability and host health [60]. Together, these studies emphasize that nutrient and vitamin exchange is not only genetically possible but functionally important for host-microbe interactions in macroalgae.

### Pathogenic Interactions

Pathogens are common in marine microbiomes, and increases in relative abundance of putative pathogens due to dysbiosis can cause diseases in the host [61, 62]. Many pathogens have a similar detrimental impact on macroalgae, such as bleaching disease in the rhodophyte *Delisea pulchra* [62], malformation disease in kelp *S. japonica* [63], and fungal diseases in the fucoid *Phyllospora comosa* [64], and the commercially valued *Saccharina japonica* [65]. Thus, monitoring variations in the epibacterial communities and functional groups across the different life stages of a macroalga can be useful to anticipate disease outbreaks.

Several bacterial taxa commonly found in macroalgal microbiomes can shift from benign or mutualistic roles to pathogenic under environmental stress, particularly elevated temperatures. In brown algae such as *Fucus vesiculosus* and *Saccharina latissima*, warming has been associated with increased abundance of *Vibrio* spp., including *V. alginolyticus* and *V. splendidus*, which can cause tissue damage and biofilm formation [66]. Similarly, *Pseudoalteromonas* and *Alteromonas*, typically considered part of the core microbiota, have been observed to contribute to tissue degradation when host-microbiome interactions are destabilized [3, 67]. In red algae, the bleaching of *D. pulchra* under heat stress has been linked to opportunistic pathogens such as *Nautella italica* R11, which disrupts the host’s chemical defenses, as well as to members of *Ruegeria* and *Thalassobius* [68, 69]. While fewer specific pathogens have been identified in green algae, similar dysbiotic shifts involving *Vibrio*, *Alteromonadaceae*, and *Flavobacteriaceae* have been reported in *Caulerpa* spp. under eutrophic and warming conditions [70]. These findings highlight how environmental stress can tip the balance of algal microbiomes, enabling opportunistic bacteria to proliferate and contribute to host decline.

## What Factors Regulate the Algal Microbiome?

The water column microbiome differs significantly from the algal microbiome across the three main groups (i.e., Chlorophyta, Rhodophyta, and Phaeophyceae), and regardless of geographical location [71, 72]. Pfister et al. [73] reported higher taxonomic and phylogenetic diversity within the kelp microbiome compared to microbiota in the seawater outside of the kelp bed, likely because in addition to environmental conditions, community structure and diversity of algal microbiomes highly depend on host specificity [30, 33, 49]. In fact, macroalgal microbiomes induce significant shifts in water column microbiota but only minor changes in the microbiomes of co-occurring algae [74], further supporting the idea that the macroalgal hosts have a strong influence on the microbiota that associate with them.

### Host Specificity

Core microbiome composition is determined by factors including host genetic relatedness [75], functional traits [76], and taxonomic level, from species identity [35] to phylum-level classifications [77]. However, the structure of bacterial communities within algal microbiomes appears to be more strongly influenced by microbial functional traits than by taxonomy, as taxonomical grouping does not always correlate with microbiome composition [20]. Differences in the microbiomes of genetically similar hosts can be due to life stage [78], morphological complexity [77, 79], tissue or thallus region [80, 81], and tissue age [12, 35, 82]. The core microbiome might be absent during early life stages of macroalgae and associate with the host organism at the adult stages [83]. Microbiome variability across different thallus regions might be due to physiological processes like oxygen metabolism [84] and chemical production [41], with epimicrobial communities generally regulated by the host’s surface metabolites [40]. Interestingly, older tissues usually have higher species abundance [79] and a more diverse microbiota [82], possibly as a result of transitory microorganisms accumulating over time [85] or increased maximum quantum yield of photosystem II [86]. Higher photosynthetic efficiency may enhance the release of organic exudates, creating a richer chemical environment that supports greater microbial diversity [87].

### Environmental Conditions

Environmental conditions can deeply affect the algal microbiome [88], often explaining spatial variability across oceans, intertidal regions [89], or between nursery and natural ecosystems [90]. The majority of studies on the impact of environmental stressors on microbiome community structure and host-microbiome interactions consider either the effects of temperature alone or the combined effects of temperature and light, carbon dioxide, or nutrient concentrations [91, 92].

### Temperature

The impact of warming on macroalgae is highly species-specific. Elevated temperatures have been shown to impair growth, reproduction, and tissue integrity in some brown algae [93, 94]. However, some species can benefit from moderate warming, depending on local adaptation and baseline thermal tolerance [95]. However, across multiple algal lineages, warming is consistently associated with microbiome dysbiosis [96, 97]. These shifts often involve an increase in opportunistic or pathogenic bacteria, weakening beneficial host-microbiome interactions and compromising algal health [30, 66].

In addition to compositional changes, elevated temperatures can disrupt key microbial functions, such as the production of antifouling or protective compounds by epiphytic bacteria. This disruption has been linked to increased susceptibility to infection and disease in both brown and red algae [69, 70]. Moreover, temperature-induced shifts in the microbiome can cascade beyond the algal host, altering trophic interactions and herbivore responses in kelp-dominated systems [14, 97]. These findings emphasize the central role of temperature in modulating not only algal physiology but also the ecological stability of host-microbe relationships.

### Light Availability

Light availability is another factor that shapes the macroalgal microbiome, with a direct impact on the host’s production of surface metabolites associated with photosynthesis, and leading to changes in epiphytic community structure [92]. Prolonged exposure to ultraviolet (UV) radiation causes a reduction in diversity and evenness of the associated bacterial community in red macroalgae [71]. Conversely, shading, often as a result of habitat transformation due to coastal urbanization, can make algal hosts, and particularly kelp species, more susceptible to colonization by fouling organisms [34]. However, the effects of light on brown algae are species-specific, since studies have shown that antifouling defense mechanisms against epibacteria are minimally altered by shading in fucoids [98]. Within the *Fucus* genus, varying levels of abiotic stressors (i.e., desiccation, temperature, and light) across the intertidal zone explain differences in the relative abundance of specific bacterial groups, suggesting that the drivers of a host’s distribution also affect its microbiome [89].

### CO_2_ Levels

Although increased CO_2_ levels do not affect the algal microbiome as much as temperature [91], they can affect the host organism’s physiology and significantly disrupt the water microbiome [14]. Ocean acidification may affect the photosynthetic efficiency of kelp, leading to bleaching and degrading tissues, potentially having indirect effects on the microbiome [61]. In giant kelp, high CO_2_ concentrations prompt the release of stress-response molecules that affect microbiome community structure, particularly targeting potentially pathogenic bacteria, to adapt to the new conditions [99]. Similarly, coralline algae rely on a tightly regulated host-microbiome interaction that guarantees the stability of the algal microbiome when exposed to high CO_2_ levels, conferring on their hosts some resilience against ocean acidification [100].

### Salinity

Salinity also impacts taxonomic composition of the microbiome while minimally altering functional profiles, likely due to the presence of high salinity-adapted bacteria that contribute to stress mitigation when exposed to a wide salinity gradient [101]. This functional stability, despite shifts in community structure, reflects a degree of redundancy that enables host acclimation across a wide salinity gradient [102].

### Nutrients

Nutrients can affect microbiome community composition directly or indirectly by driving physiological changes in the host macroalgae. Nutrient enrichment affects the taxonomic and functional structure of kelp-associated bacteria, which have shown a low community resistance index throughout the thallus [103]. Rot diseases that disrupt the bacterial community of brown algae in favor of degraders that alter community functions, and in turn exacerbate the disease, have been attributed to seawater nutrient levels [65]. Both phosphorus and nitrogen concentrations are strongly correlated with the relative abundance of Bacteroidetes and *Vibrio* species in green [104] and brown [105] algae, respectively. These microbiome responses are potentially linked to higher algal dissolved organic carbon (DOC) and dissolved organic matter (DOM) release rates under high nutrient availability and the subsequent consumption by these bacteria [67]. Nitrogen availability modulates the role of bacteria-kelp interactions in enhancing kelp growth, although its regulatory effect depends on the genetic background of the host [106]. Apart from changes in the water’s physicochemical properties, the presence of epiphytic bryozoans also alters the kelp microbiome, potentially causing dysbiosis and the proliferation of opportunistic pathogens that can have a detrimental effect on the host’s health and productivity [107].

### Biogeography

At the interface between host and environmental factors that influence microbiome composition are spatial distribution and seasonal variation. The relative influence of geographic location on algal microbiome structure depends on phylum and species, with a generally greater influence in red than green or brown algae [104]. While kelp microbiomes exhibit intraspecific differences among largely disconnected geographical areas, some remain relatively stable within regions [37]. In fact, several studies have found a very high correspondence in bacterial communities among sympatric species’ microbiomes regardless of geographical location [97, 108]. Notably, microbiome community structure, and particularly bacterial composition, can greatly vary at small spatial scales (< 100 m) [72, 109]. Roth-Schulze et al. [110] described core functional genes in the microbiome of *Ulva* spp. spanning a large geographical area and suggesting functional biogeography might be more strongly linked to microbiome composition than geographical distance. Another study reported similar structure and alpha diversity in the microbiome of *F. vesiculosus* across a latitudinal gradient [111]. These data suggest that spatial distribution is secondary to host identity in regulating algal microbiomes.

## Does the Microbiome Exacerbate or Mitigate the Impact of Climate Change on Its Algal Host?

Climate change poses a complex and growing threat to marine macroalgae, not only by directly affecting their physiology, but also through indirect effects on their microbiomes. Key stressors such as ocean warming, acidification, nutrient enrichment, and increased irradiance can impair macroalgal metabolism and growth, ultimately influencing population health and ecosystem function [51]. For instance, elevated temperatures may reduce photosynthetic efficiency, damage cellular structures, and affect reproductive timing in various macroalgal groups [91]. Acidification may inhibit calcification processes and alter nutrient uptake, while eutrophication can cause nutrient imbalances that further affect macroalgal hosts [96, 112]. These stressors, when combined, can severely disrupt the functional equilibrium of the holobiont.

Beyond the direct effects on algal physiology, climate change can destabilize the intricate relationships between macroalgae and their associated microbiota, leading to a state of microbial imbalance often associated with negative health outcomes known as dysbiosis. As seen in corals, warming can lead to shifts in microbial community composition that favor opportunistic or pathogenic taxa, triggering bleaching or disease outbreaks [113]. A similar pattern has been observed in red algae such as *D. pulchra*, where thermal stress resulted in microbiota restructuring and increased pathogenicity, ultimately causing bleaching and tissue loss [114]. In addition to the increase in opportunistic pathogens, there is a risk of losing beneficial or essential symbionts that contribute to host resilience through nutrient cycling, stress protection, and immune regulation [115, 116]. An example of this is the inhibition of key microbiome functions in rhodoliths and subsequent resilience loss due to ocean acidification [100]. Thus, climate-driven microbiome disruption can simultaneously amplify harmful microbial interactions and compromise essential mutualistic functions.

Despite the growing concern over the destabilizing effects of climate stress, the joint effects of multiple environmental stressors on algal microbiomes remain understudied compared to free-living aquatic microbiota. Multiple-stressor effects on the microbiome have mostly been evaluated in descriptive studies rather than controlled mesocosms or laboratory conditions. The few studies that assessed the joint effects of temperature and other drivers on macroalgae found both additive [91] and synergistic [14] effects with CO_2_, synergistic effects with light [92], and additive effects with nutrient stress [96, 103]. Minich et al. [14] observed stimulated kelp growth and an increase in microbiome diversity under the combined impacts of high temperature and CO_2_ levels, consistent with a broader trend observed in other marine hosts where environmental stress leads to a diversification of the microbiome [16, 112, 117]. These shifts may reflect both adaptive microbial responses and increased holobiont instability, with more targeted research needed to disentangle the two.

In this context, an important emerging question is whether the microbiome can also mitigate or buffer the negative impacts of climate change on macroalgae. Some microbial taxa provide functions that help the host tolerate abiotic stress, such as enhancing nitrogen uptake, producing antioxidants, or modulating hormone signaling pathways [115, 116]. For example, specific bacterial taxa have been shown to improve thermal tolerance or support recovery after bleaching events [118], akin to “beneficial probiotics” in corals and terrestrial plants. Bonthond et al. [117] found that non-native populations of *Gracilaria vermiculophylla* exhibited more stable microbiota under thermal stress than native ones, suggesting that invasion processes may select for microbiome configurations that are more resilient or protective. Similarly, Molnar et al. [112] showed that shifts in microbial functional traits under combined stress conditions could help maintain host performance through metabolic compensation. However, these protective capacities appear to be context-dependent, highlighting the need for a mechanistic understanding of host-microbe interactions under variable environmental conditions.

The responses of algal microbiomes under future climate scenarios are uncertain, and identifying their regulatory factors (i.e., the molecular, physiological, and ecological mechanisms that shape microbial community dynamics in changing environments) remains a challenge [119]. Studying climate impacts on algal microbiomes is not only crucial for predicting ecosystem responses, but also provides broader insights into holobiont dynamics, particularly under new environmental scenarios [120]. Many of the traits that facilitate macroalgal resilience to climate change, such as microbiome flexibility, compositional stability, and functional redundancy, are also central to the success of biological invasions [117]. Just as invasive macroalgal species must rapidly adapt to unfamiliar environments, climate change imposes sudden and shifting conditions that challenge the stability of host-microbiome systems. Exploring this overlap may help identify general principles of holobiont adaptability and guide strategies for ecosystem restoration, biocontrol, or assisted adaptation.

Taken together, these findings underscore the importance of integrating microbiome research into marine climate resilience frameworks. Future studies should aim for multi-factorial experimental designs, broader taxonomic and geographic coverage, and a deeper focus on microbial functionality rather than taxonomy alone. These approaches will help clarify the dual role of the microbiome, conferring resilience or exacerbating stress, in shaping the future of macroalgal ecosystems.

## The Role of the Microbiome in the Invasive Potential of Macroalgae

Despite the growing evidence that the microbiome strongly influences invasive potential (i.e., the development, spread, and potential impact on native ecosystems) of non-native macroalgae, very little is known about the underlying dynamics and processes. The exact mechanisms through which the microbiomes of invasive seaweeds confer on them a competitive advantage over native species differ among species. However, there are some similarities across phyla, and even with invasive corals [17]. Generally speaking, the successful introduction of invasive brown algae to new environments is attributed to the high diversity and successional rate of their associated microbiota. For example, the microbiome of *Sargassum muticum* shows high spatiotemporal and tissue-specific variability, supporting the idea that microbiota associated with invasive species are very dynamic and adaptable, which can potentially enhance their ecological success and competitive advantage [85]. Moreover, studies on the invasive *Sargassum horneri* suggest that the host organism may alter its surrounding microbial environment, exacerbating the impact of other external disturbances and delaying the recovery of native kelp populations [51].

The flexibility of host-microbiome associations in non-native seaweeds has also been reported in red and green algae. For instance, microbial interactions are crucial for understanding the spread of *Asparagopsis* spp., whose microbiome is influenced by both the host and environmental factors, possibly contributing to its success in new ecosystems [49]. Similarly, studies on the microbiomes of *Asparagopsis* spp. [121] and *Gracilaria* spp. [122] show that host promiscuity (i.e., its ability to form relationships with a diverse number of species within the microbiome) can facilitate invasion processes by enhancing ecological adaptability and allowing these species to better adjust to new environments. Moreover, a recent experimental study found that microbiome beta diversity increased more in native than non-native *G. vermiculophylla* populations under thermal stress, suggesting that invasion processes may select for traits that make non-native hosts more stable and resilient to microbial disruptions under stress [117].

Research on the microbiome of non-native green algae has mostly focused on invasive *Caulerpa taxifolia*, and particularly its spread in the Mediterranean. Studies have found a strong alignment between the algal host’s genomic and endophytic microbial diversities, reinforcing the holobiont concept as the core unit of invasion and spread [70]. In fact, *C. taxifolia*’s recent regression in the Mediterranean might be linked to the holobiont’s low genetic diversity, highlighting the potential adaptive advantages provided by metabolically active bacterial communities. In addition to microbiome flexibility, some environmental stressors can strengthen the resistance and resilience of invasive green algae. For example, nutrient enrichment contributes to the proliferation of *Caulerpa* spp. through alterations in its associated bacterial communities [76, 123]. These findings highlight the adaptive potential of invasive seaweeds, with microbiota that may provide them with ecological advantages, including stress tolerance and enhanced growth.

Characterizing bacterial communities that support the proliferation of invasive macroalgae can be used to trace the origin of invasion and implement more informed management strategies [49]. Comparing the microbiomes of co-occurring native and non-native macroalgae can help assess invasion risk, as microbiome transfer may indicate increased potential for invasive success [124]. Similarly, understanding the microbiome of native macroalgae could help identify microbial factors that enhance resilience to both environmental stressors and invasions [9]. By focusing on the functional roles within the microbiome and understanding host-microbiome interactions, future research could help develop strategies to anticipate and mitigate the impacts of invasive species.

## Applications of the Algal Microbiome in Sustainable Industrial Development

The potential of the algal microbiome for industrial applications is immense. Specifically, it can be used to improve the sustainability and efficiency of seaweed farming, either for consumption or for the development of bio-stimulants that offer an environmentally friendly alternative to the chemicals traditionally used in agricultural practices [54]. Identification of ecological and functional core microbiome taxa, particularly polysaccharide depolymerizing and growth-promoting groups, can be useful to develop new aquaculture strategies for commercially valuable seaweeds. Several studies have shown that specific bacterial strains can significantly increase the biomass of *Ulva* spp. [53, 125], highlighting the potential of microbiome manipulation to enhance seaweed productivity.

The use of beneficial microbes to reduce pathogens can ensure healthier and more robust seaweed crops for both environmental and industrial use. The “microbial gardening” approach has been shown to attract beneficial bacteria that provide protection against pathogens in *Agarophyton vermiculophyllum*, thereby enhancing seaweed health [126]. A combination of reductionist and systems biology (i.e., breaking down the components of the system and then putting them together for a comprehensive network analysis) might be an effective approach in microbiome engineering, with potential to increase the adaptability of macroalgae to stress [120]. Manipulating algal microbiomes across multiple species could make seaweed aquaculture more efficient, contributing to carbon capture efforts and sustainable resource production [127].

Understanding and manipulating microbial interactions within the microbiome can be used to enhance algal lipid production, lowering operational costs and improving the feasibility of biofuels derived from algae [128]. For instance, microorganisms capable of breaking down complex carbohydrates like agarase and carrageenase hold promise for converting seaweed biomass into bioethanol [129]. Moreover, host-bacteria associations could be harnessed for industrial benefits, as they regulate the microbiome’s functional roles of supporting the health and growth of seaweeds [67]. The enzymatic potential of seaweed-associated microbes opens avenues for applications in multiple sectors, and with reduced environmental impacts relative to conventional biofuel production.

## Future Directions in Microbiome Research

Future microbiome research should prioritize determining whether the host or the microbiota respond first to global stressors, and studying the shifts in host-microbiome dynamics and their underlying infochemical mechanisms in response to climate change [130]. Despite being secondary to host specificity in shaping microbiota, seasonal fluctuations in environmental factors shape the presence of infochemicals in macroalgae and therefore must be carefully considered in microbiome studies [41]. In addition to descriptive studies across natural gradients of environmental drivers, experimental manipulations are needed to shed light on how holobionts will interact with their environment under future climate change scenarios [115]. Rather than focusing on the regulating effect of a single factor, studies should include multiple-stressor and multi-factorial experimental designs that consider host genetics, morphology, geography, and water quality parameters, all of which are essential in structuring the microbiome of foundational seaweeds [131].

Besides direct species restoration, maintaining a functional microbiota may be the key to the success of microbiome-assisted restoration of marine habitats [132]. To achieve this, there is an urgent need to catalog microbiome genes and their functions using meta-omic approaches to link host and microbiome gene expression [115]. Furthermore, the possibility of forecasting abrupt microbiome events by applying classic ecological concepts to diverse microbial systems [133] could be extremely useful to anticipate macroalgal responses to climate change, including harmful algal blooms. Expanding microbiome research to kelp species in the Southern Hemisphere will also provide a more comprehensive understanding of how microbiota vary across different ecological contexts and environmental gradients, helping to inform conservation and management strategies.

Future research should also explore the role of microbiomes in developing sustainable macroalgae farming strategies. Countries such as China, Indonesia, Norway, and Chile are major producers of farmed macroalgae species like *Saccharina*, *Gracilaria*, and *Macrocystis* [134, 135], facing challenges including disease, environmental stress, and reduced productivity [136]. Microbiome manipulation, using techniques such as microbial inoculation or the selection of microbiome-compatible host genotypes, can be an extremely effective approach to support host health, increase resilience, and enhance yield [67, 127]. These practices could help overcome issues in large-scale seaweed farming and promote environmentally sustainable aquaculture practices.

Moving forward, microbiome research should be centered around comprehensive experimental studies integrating chemical ecology and drawing lessons from coral microbiome manipulation research [137] to enhance long-term resilience against global stressors, particularly in foundational seaweeds like kelp forests. The detection of early warning indicators in microbiomes anticipating the potential ecological impact of non-native macroalgae can provide valuable insights into how to manage and control invasions. Finally, the application of new technologies, such as high-throughput sequencing and metabolomic profiling, offers unprecedented opportunities to explore the full complexity of kelp microbiomes.

## Conclusion

Macroalgal microbiomes are central to the health and resilience of marine ecosystems, yet significant knowledge gaps remain regarding their roles in host adaptation, ecological dynamics, and responses to global change. While research has highlighted their potential in informing conservation and restoration, there is an urgent need for a more critical understanding of microbial function, biogeographic variability, and long-term ecosystem impacts. Integrating microbiome studies with climate science, invasion biology, and ecosystem management, especially in underrepresented regions and taxa, will be essential for developing predictive and actionable strategies. While algal microbiome research holds great promise for predicting and mitigating the effects of climate change and invasive species, embracing new tools and tackling ecologically relevant mechanistic and applied questions will be essential to advancing this field.

## Data Availability

No datasets were generated or analysed during the current study.

## References

[1] Berg G, Rybakova D, Fischer D, Cernava T, Vergès MCC, Charles T et al (2020) Microbiome definition re-visited: old concepts and new challenges. Microbiome 8:103. 10.1186/s40168-020-00875-0 32605663 10.1186/s40168-020-00875-0 PMC7329523

[2] Marchesi JR, Ravel J (2015) The vocabulary of microbiome research: a proposal. Microbiome 3:1–3 26229597 10.1186/s40168-015-0094-5 PMC4520061

[3] Egan S, Harder T, Burke C, Steinberg P, Kjelleberg S, Thomas T (2013) The seaweed holobiont: understanding seaweed–bacteria interactions. FEMS Microbiol Rev 37:462–476 23157386 10.1111/1574-6976.12011

[4] Astudillo-García C, Bell JJ, Webster NS, Glasl B, Jompa J, Montoya JM et al (2017) Evaluating the core microbiota in complex communities: a systematic investigation. Environ Microbiol 19:1450–1462 28078754 10.1111/1462-2920.13647

[5] Banerjee S, Schlaeppi K, van der Heijden MGA (2018) Keystone taxa as drivers of microbiome structure and functioning. Nat Rev Microbiol 16:567–576 29789680 10.1038/s41579-018-0024-1

[6] Liu Y, Geng Y, Jiang Y, Li P, Li Y, Zhang Z (2025) Global microbial community biodiversity increases with antimicrobial toxin abundance of rare taxa. ISME J 19(1):012wraf 10.1093/ismejo/wraf012 PMC11822679 39849986

[7] Tourneroche A, Lami R, Burgaud G, Domart-Coulon I, Li W, Gachon C et al (2020) The bacterial and fungal microbiota of *Saccharina latissima* (Laminariales, Phaeophyceae). Front Mar Sci 7:587566

[8] Bordenstein SR, Theis KR (2015) Host biology in light of the microbiome: ten principles of holobionts and hologenomes. PLoS Biol 13:e1002226 26284777 10.1371/journal.pbio.1002226 PMC4540581

[9] McGrath AH, Lema K, Egan S, Wood G, Gonzalez SV, Kjelleberg S et al (2024) Disentangling direct vs indirect effects of microbiome manipulations in a habitat-forming marine holobiont. Npj Biofilms Microbiomes 10:33 38553475 10.1038/s41522-024-00503-x PMC10980776

[10] Vandenkoornhuyse P, Quaiser A, Duhamel M, le Van A, Dufresne A (2015) The importance of the microbiome of the plant holobiont. New Phytol 206:1196–1206 25655016 10.1111/nph.13312

[11] Pearman WS, Morales SE, Vaux F, Gemmell NJ, Fraser CI (2023) Differences in density: taxonomic but not functional diversity in seaweed microbiomes affected by an earthquake. bioRxiv 2022–23.

[12] Lemay MA, Chen MY, Mazel F, Hind KR, Starko S, Keeling PJ et al (2021) Morphological complexity affects the diversity of marine microbiomes. ISME J 15:1372–1386 33349654 10.1038/s41396-020-00856-z PMC8115056

[13] Wainwright BJ, Millar T, Bowen L, Semon L, Hickman KJE, Lee JN et al (2023) The core mangrove microbiome reveals shared taxa potentially involved in nutrient cycling and promoting host survival. Environ Microbiome 18:47 37264467 10.1186/s40793-023-00499-5 PMC10236742

[14] Minich JJ, Morris MM, Brown M, Doane M, Edwards MS, Michael TP et al (2018) Elevated temperature drives kelp microbiome dysbiosis, while elevated carbon dioxide induces water microbiome disruption. PLoS ONE 13:e0192772 29474389 10.1371/journal.pone.0192772 PMC5825054

[15] Egan S, Gardiner M (2016) Microbial dysbiosis: rethinking disease in marine ecosystems. Front Microbiol 7:991 27446031 10.3389/fmicb.2016.00991 PMC4914501

[16] Zaneveld JR, McMinds R, Vega Thurber R (2017) Stress and stability: applying the Anna Karenina principle to animal microbiomes. Nat Microbiol 2:1–8 10.1038/nmicrobiol.2017.121 28836573

[17] Girija GK, Tseng LC, Chen YL, Meng PJ, Hwang JS, Ho YN (2023) Microbiome variability in invasive coral (Tubastraea aurea) in response to diverse environmental stressors. Front Mar Sci 10:1234137

[18] Bell JJ, Bennett HM, Rovellini A, Webster NS (2018) Sponges to be winners under near-future climate scenarios. Bioscience 68(12):955–968

[19] Lu DC, Wang FQ, Amann RI, Teeling H, Du ZJ (2023) Epiphytic common core bacteria in the microbiomes of co-located green (Ulva), brown (Saccharina), and red (Grateloupia, Gelidium) macroalgae. Microbiome 11:126 37264413 10.1186/s40168-023-01559-1 PMC10233909

[20] Florez JZ, Camus C, Hengst MB, Buschmann AH (2017) A functional perspective analysis of macroalgae and epiphytic bacterial community interaction. Front Microbiol 8:2561 29312241 10.3389/fmicb.2017.02561 PMC5743738

[21] Singh D, Valdenegro-Toro M. (2021). The marine debris dataset for forward-looking sonar semantic segmentation. Proceedings of the Ieee/Cvf International Conference on Computer Vision, 3741–3749.

[22] Ferreira JCN, Bergo NM, Tura PM, Chuqui MG, Brandini FP, Jovane L, Pellizari VH (2022) Abundance and microbial diversity from surface to deep water layers over the Rio Grande Rise South Atlantic. Prog Oceanogr 201:102736

[23] Bourne DG, Morrow KM, Webster NS (2016) Insights into the coral microbiome: underpinning the health and resilience of reef ecos**y**stems. Annu Rev Microbiol 70(1):317–340 27482741 10.1146/annurev-micro-102215-095440

[24] Van Oppen MJH, Blackall LL (2019) Coral microbiome dynamics, functions and design in a changing world. Nat Rev Microbiol 17(9):557–567 31263246 10.1038/s41579-019-0223-4

[25] Thomas T, Moitinho-Silva L, Lurgi M, Björk JR, Easson C, Astudillo-García C et al (2016) Diversity, structure and convergent evolution of the global sponge microbiome. Nat comm 7(1):11870 10.1038/ncomms11870 PMC4912640 27306690

[26] Pita L, Rix L, Slaby BM, Franke A, Hentschel U (2018) The sponge holobiont in a changing ocean: from microbes to ecosystems. Microbiome 6(1):46 29523192 10.1186/s40168-018-0428-1 PMC5845141

[27] Bulgarelli D, Schlaeppi K, Spaepen S, van Themaat EVL, Schulze-Lefert P (2013) Structure and functions of the bacterial microbiota of plants. Annu Rev Plant Biol 64(1):807–838 23373698 10.1146/annurev-arplant-050312-120106

[28] Turner TR, James EK, Poole PS (2013) The plant microbiome. Genome Biol 14:1–10 10.1186/gb-2013-14-6-209 PMC3706808 23805896

[29] Marzinelli EM, Thomas T, Vadillo Gonzalez S, Egan S, Steinberg PD (2024) Seaweeds as holobionts: Current state, challenges, and potential applications. J Phycol 60(4):785–796 39047050 10.1111/jpy.13485

[30] Campbell AH, Marzinelli EM, Gelber J, Steinberg PD (2015) Spatial variability of microbial assemblages associated with a dominant habitat-forming seaweed. Front Microbiol 6:230 25859245 10.3389/fmicb.2015.00230 PMC4374473

[31] Aguirre EG, Schwartzman JA (2024) Metagenome-assembled genomes of *Macrocystis*-associated bacteria. Microbiol Resour Announc 13:e00715-e724 39436062 10.1128/mra.00715-24 PMC11556038

[32] Calderón MS, Bustamante DE, Rosselli R (2024) Functional prediction based on 16S rRNA metagenome data from bacterial microbiota associated with macroalgae from the Peruvian coast. Mar Drugs 22(1):1–19 10.1038/s41598-024-69538-6 PMC11316746 39127849

[33] Theirlynck T, Mendonça IRW, Engelen AH, Bolhuis H, Collado-Vides L, van Tussenbroek BI et al (2023) Diversity of the holopelagic *Sargassum* microbiome from the Great Atlantic Sargassum Belt to coastal stranding locations. Harmful Algae 122:102369 36754458 10.1016/j.hal.2022.102369

[34] Marzinelli EM, Qiu Z, Dafforn KA, Johnston EL, Steinberg PD, Mayer-Pinto M (2018) Coastal urbanization affects microbial communities on a dominant marine holobiont. Npj Biofilms Microbiomes 4:1 29367878 10.1038/s41522-017-0044-z PMC5772048

[35] Weigel BL, Pfister CA (2019) Successional dynamics and seascape-level patterns of microbial communities on the canopy-forming kelps *Nereocystis luetkeana* and *Macrocystis pyrifera*. Front Microbiol 10:346 30863387 10.3389/fmicb.2019.00346 PMC6399156

[36] Saha M, Dittami SM, Chan CX, Raina J, Stock W, Ghaderiardakani F et al (2024) Progress and future directions for seaweed holobiont research. New Phytol 244(2):364–376 39137959 10.1111/nph.20018

[37] Phelps CM, McMahon K, Bissett A, Bernasconi R, Steinberg PD, Thomas T et al (2021) The surface bacterial community of an Australian kelp shows cross-continental variation and relative stability within regions. FEMS Microbiol Ecol 97:fiab089 34156064 10.1093/femsec/fiab089

[38] Ochoa-Sánchez M, Gomez EPA, Ramírez-Fernández L, Eguiarte LE, Souza V (2023) Current knowledge of the Southern Hemisphere marine microbiome in eukaryotic hosts and the Strait of Magellan surface microbiome project. PeerJ 11:e15978 37810788 10.7717/peerj.15978 PMC10557944

[39] Wang J, Tang X, Mo Z, Mao Y (2022) Metagenome-assembled genomes from *Pyropia haitanesis* microbiome provide insights into the potential metabolic functions to the seaweed. Front Microbiol 13:857901 35401438 10.3389/fmicb.2022.857901 PMC8984609

[40] Othmani A, Briand JF, Ayé M, Molmeret M, Culioli G (2016) Surface metabolites of the brown alga *Taonia atomaria* have the ability to regulate epibiosis. Biofouling 32:801–813 27353006 10.1080/08927014.2016.1198954

[41] Paix B, Carriot N, Barry-Martinet R, Greff S, Misson B, Briand JF et al (2020) A multi-omics analysis suggests links between the differentiated surface metabolome and epiphytic microbiota along the thallus of a Mediterranean seaweed holobiont. Front Microbiol 11:494 32269559 10.3389/fmicb.2020.00494 PMC7111306

[42] Burgunter-Delamare B, Tanguy G, Legeay E, Boyen C, Dittami SM (2022) Effects of sampling and storage procedures on 16S rDNA amplicon sequencing results of kelp microbiomes. Mar Genomics 63:100944 35299055 10.1016/j.margen.2022.100944

[43] Lachnit T, Fischer M, Künzel S, Baines JF, Harder T (2013) Compounds associated with algal surfaces mediate epiphytic colonization of the marine macroalga *Fucus vesiculosus*. FEMS Microbiol Ecol 84(2):411–420 23311942 10.1111/1574-6941.12071

[44] Laiolo E, Alam I, Uludag M, Jamil T, Agusti S, Gojobori T et al (2024) Metagenomic probing toward an atlas of the taxonomic and metabolic foundations of the global ocean genome. Front Sci 1:1038696

[45] Li J, Weinberger F, de Nys R, Thomas T, Egan S (2023) A pathway to improve seaweed aquaculture through microbiota manipulation. Trends Biotech 41(4):545–556 10.1016/j.tibtech.2022.08.003 36089422

[46] Wichard T (2015) Exploring bacteria-induced growth and morphogenesis in the green macroalga order Ulvales (Chlorophyta). Front Plant Sci 6:86 25784916 10.3389/fpls.2015.00086 PMC4347444

[47] Goecke F, Labes A, Wiese J, Imhoff JF (2010) Chemical interactions between marine macroalgae and bacteria. Mar Ecol Prog Ser 409:267–299

[48] Egan S, Fernandes ND, Kumar V, Gardiner M, Thomas T (2014) Bacterial pathogens, virulence mechanism and host defense in marine macroalgae. Environ Microbiol 16(4):925–938 24112830 10.1111/1462-2920.12288

[49] Aires T, Serrão EA, Engelen AH (2016) Host and environmental specificity in bacterial communities associated to two highly invasive marine species (genus *Asparagopsis*). Front Microbiol 7:559 27148239 10.3389/fmicb.2016.00559 PMC4839258

[50] Dogs M, Wemheuer B, Wolter L, Bergen N, Daniel R, Simon M et al (2017) *Rhodobacteraceae* on the marine brown alga *Fucus spiralis* are abundant and show physiological adaptation to an epiphytic lifestyle. Syst Appl Microbiol 40:370–382 28641923 10.1016/j.syapm.2017.05.006

[51] Morris MM, Dinsdale EA. (2019) Microbiome warfare: The potential of microbes from invasive alga *Sargassum horneri* to interfere with recovery of native kelp. ESA Annu Meet (August 11–16).

[52] Michelou VK, Caporaso JG, Knight R, Palumbi SR (2013) The ecology of microbial communities associated with *Macrocystis pyrifera*. PLoS ONE 8:e67480 23840715 10.1371/journal.pone.0067480 PMC3686729

[53] Ghaderiardakani F, Ulrich JF, Barth E, Quartino ML, Wichard T (2024) Algal growth and morphogenesis-promoting factors released by cold-adapted bacteria contribute to the resilience and morphogenesis of the seaweed *Ulva* (Chlorophyta) in Antarctica (Potter Cove). J Plant Growth Regul 1–18.

[54] Qu T, Zhao X, Guan C, Hou C, Chen J, Zhong Y et al (2023) Structure-function covariation of phycospheric microorganisms associated with the typical cross-regional harmful macroalgal bloom. Appl Environ Microbiol 89:e01815-e1822 36533927 10.1128/aem.01815-22 PMC9888261

[55] Vigil BE, Ascue F, Ayala RY, Murúa P, Calderon MS, Bustamante DE (2024) Functional prediction based on 16S rRNA metagenome data from bacterial microbiota associated with macroalgae from the Peruvian coast. Sci Rep 14:18577 39127849 10.1038/s41598-024-69538-6 PMC11316746

[56] Hochroth A, Pfister CA (2024) Ammonification by kelp-associated microbes increases ammonium availability. PLoS ONE 19:e0296622 38551914 10.1371/journal.pone.0296622 PMC10980195

[57] Weigel BL, Miranda KK, Fogarty EC, Watson AR, Pfister CA (2022) Functional insights into the kelp microbiome from metagenome-assembled genomes. mSystems 7:e01422-21 35642511 10.1128/msystems.01422-21 PMC9238374

[58] Croft MT, Warren MJ, Smith AG (2006) Algae need their vitamins. Eukaryotic cell 5(8):1175–1183 16896203 10.1128/EC.00097-06 PMC1539151

[59] Kazamia E, Czesnick H, van Nguyen TT, Croft MT, Sherwood E, Sasso S et al (2012) Mutualistic interactions between vitamin B12-dependent algae and heterotrophic bacteria exhibit regulation. Environ Microbiol 14(6):1466–1476 22463064 10.1111/j.1462-2920.2012.02733.x

[60] Zhao J, Nair S, Zhang Z, Wang Z, Jiao N, Zhang Y (2024) Macroalgal virosphere assists with host–microbiome equilibrium regulation and affects prokaryotes in surrounding marine environments. ISME J 18:wrae083 38709876 10.1093/ismejo/wrae083 PMC11126160

[61] Qiu Z, Coleman MA, Provost E, Campbell AH, Kelaher BP, Dalton SJ et al (2019) Future climate change is predicted to affect the microbiome and condition of habitat-forming kelp. Proc R Soc B 286:20181887 30963929 10.1098/rspb.2018.1887 PMC6408609

[62] Kumar V, Zozaya-Valdes E, Kjelleberg S, Thomas T, Egan S (2016) Multiple opportunistic pathogens can cause a bleaching disease in the red seaweed *Delisea pulchra*. Environ Microbiol 18:3962–3975 27337296 10.1111/1462-2920.13403

[63] Yan Y, Wang S, Liu K, Mo Z, Yang H, Rong X et al (2023) Divergence of epibacterial community assemblage correlates with malformation disease severity in *Saccharina japonica* seedlings. Front Mar Sci 10:1089349

[64] Ferrari J, Goncalves P, Campbell AH, Sudatti DB, Wood GV, Thomas T et al (2021) Molecular analysis of a fungal disease in the habitat-forming brown macroalga *Phyllospora comosa* (*Fucales*) along a latitudinal gradient. J Phycol 57:1504–1516 33942303 10.1111/jpy.13180

[65] Ma C, Peng C, Fu L, Ren C, Liu X, Liu Z et al (2024) Phycosphere bacterial disturbance of *Saccharina japonica* caused by white rot disease relates to seawater nutrients. Environ Sci Pollut Res 31:37245–37255 10.1007/s11356-024-33707-x 38767795

[66] Stratil SB, Neulinger SC, Knecht H, Friedrichs AK, Wahl M (2013) Temperature-driven shifts in the epibiotic bacterial community composition of the brown macroalga *Fucus vesiculosus*. Microbiology Open 2(2):338–349 23568841 10.1002/mbo3.79 PMC3633357

[67] Hollants J, Leliaert F, de Clerck O, Willems A (2013) What we can learn from sushi: a review on seaweed–bacterial associations. FEMS Microbiol Ecol 83:1–16 22775757 10.1111/j.1574-6941.2012.01446.x

[68] Fernandes N, Case RJ, Longford SR, Seyedsayamdost MR, Steinberg PD, Kjelleberg S et al (2011) Genomes and virulence factors of novel bacterial pathogens causing bleaching disease in the marine red alga Delisea pulchra. PLoS ONE 6(12):e27387 22162749 10.1371/journal.pone.0027387 PMC3230580

[69] Zozaya-Valdes E, Egan S, Thomas T (2015) A comprehensive analysis of the microbial communities of healthy and diseased marine macroalgae and the detection of known and potential bacterial pathogens. Front Microbiol 6:146 25759688 10.3389/fmicb.2015.00146 PMC4338804

[70] Dittami SM, Arboleda E, Auguet JC, et. al. (2020) A community perspective on the concept of marine holobionts: current status, challenges, and future directions. PCI Ecology 10.7717/peerj.10911 PMC7916533 33665032

[71] Dobretsov S, Véliz K, Romero MS, Tala F, Thiel M (2021) Impact of UV radiation on the red seaweed *Gelidium lingulatum*and its associated bacteria. Eur J Phycol 56:129–141

[72] King NG, Moore PJ, Thorpe JM, Smale DA (2023) Consistency and variation in the kelp microbiota: patterns of bacterial community structure across spatial scales. Microb Ecol 85:1265–1275 35589992 10.1007/s00248-022-02038-0

[73] Pfister CA, Altabet MA, Weigel BL (2019) Kelp beds and their local effects on seawater chemistry, productivity, and microbial communities. Ecology 100:e02798 31233610 10.1002/ecy.2798

[74] Chen MY, Parfrey LW (2018) Incubation with macroalgae induces large shifts in water column microbiota, but minor changes to the epibiota of co-occurring macroalgae. Mol Ecol 27:1966–1979 29524281 10.1111/mec.14548

[75] Vadillo Gonzalez S, Vranken S, Coleman MA, Wernberg T, Steinberg PD, Marzinelli EM (2023) Host genotype and microbiome associations in co-occurring clonal and non-clonal kelp, *Ecklonia radiata*. Mol Ecol 32:4584–4598 37332135 10.1111/mec.17056

[76] Morrissey KL, Çavaş L, Willems A, de Clerck O (2019) Disentangling the influence of environment, host specificity and thallus differentiation on bacterial communities in siphonous green seaweeds. Front Microbiol 10:717 31024496 10.3389/fmicb.2019.00717 PMC6460459

[77] Kuba GM, Spalding HL, Hill-Spanik KM, Fullerton H (2021) Microbiota-macroalgal relationships at a Hawaiian intertidal bench are influenced by macroalgal phyla and associated thallus complexity. mSphere 6:e0101128 10.1128/mSphere.00665-21 PMC8550217 34550007

[78] Lemay MA, Martone PT, Hind KR, Lindstrom SC, Wegener Parfrey L (2018) Alternate life history phases of a common seaweed have distinct microbial surface communities. Mol Ecol 27:3555–3568 30055017 10.1111/mec.14815

[79] Lemay MA, Davis KM, Martone PT, Parfrey LW (2021) Kelp-associated microbiota are structured by host anatomy. J Phycol 57:1119–1130 33749821 10.1111/jpy.13169

[80] Ihua MW, FitzGerald JA, Guihéneuf F, Jackson SA, Claesson MJ, Stengel DB et al (2020) Diversity of bacterial populations associated with different thallus regions of the brown alga *Laminaria digitata*. PLoS ONE 15:e0242675 33237941 10.1371/journal.pone.0242675 PMC7688147

[81] Burgunter-Delamare B, Rousvoal S, Legeay E, Tanguy G, Fredriksen S, Boyen C et al. (2022) The *Saccharina latissima* microbiome: algal tissue matters more than region, season, and physiology. bioRxiv 2022–26. 10.3389/fmicb.2022.1050939 PMC9858215 36687663

[82] Lin JD, Lemay MA, Parfrey LW (2018) Diverse bacteria utilize alginate within the microbiome of the giant kelp *Macrocystis pyrifera*. Front Microbiol 9:1914 30177919 10.3389/fmicb.2018.01914 PMC6110156

[83] Park J, Schenk S, Davis K, Clark J, Parfrey LW (2023) Exploring the impact of microbial manipulation on the early development of kelp (*Saccharina latissima*) using an ecological core microbiome framework. bioRxiv 2012–23.

[84] Weigel BL, Pfister CA (2021) Oxygen metabolism shapes microbial settlement on photosynthetic kelp blades compared to artificial kelp substrates. Environ Microbiol Rep 13:176–184 33372322 10.1111/1758-2229.12923

[85] Aires T, Kläui A, Engelen A (2023) Regional microbiome differentiation of the invasive *Sargassum muticum* (*Fucales*, *Phaeophyceae*) follows the generalist host hypothesis across the North East Atlantic. Eur J Phycol 58:268–283

[86] Esaian S, Bui A, DiFiore BP, Peters JR, Lepori-Bui M, Husted K et al. (2024) Maturing giant kelp develop depth-specific microbiomes. bioRxiv 2024.

[87] Haas AF, Nelson CE, Rohwer F, Wegley-Kelly L, Quistad SD, Carlson CA et al (2013) Influence of coral and algal exudates on microbially mediated reef metabolism. PeerJ 1:e108 23882445 10.7717/peerj.108 PMC3719129

[88] Pearman WS, Duffy GA, Liu XP, Gemmell NJ, Morales SE, Fraser CI (2024) Macroalgal microbiome biogeography is shaped by environmental drivers rather than geographical distance. Ann Bot 133:169–182 37804485 10.1093/aob/mcad151 PMC10921836

[89] Quigley CTC, Capistrant-Fossa KA, Morrison HG, Johnson LE, Morozov A, Hertzberg VS et al (2020) Bacterial communities show algal host (Fucus spp.)/zone differentiation across the stress gradient of the intertidal zone. Front Microbiol 11:563118 33072025 10.3389/fmicb.2020.563118 PMC7541829

[90] Davis KM, Zeinert L, Byrne A, Davis J, Roemer C, Wright M et al (2023) Successional dynamics of the cultivated kelp microbiome. J Phycol 59:538–551 37005360 10.1111/jpy.13329

[91] Mensch B, Neulinger SC, Graiff A, Pansch A, Künzel S, Fischer MA et al (2016) Restructuring of epibacterial communities on *Fucus vesiculosus* forma *mytili* in response to elevated pCO_2_ and increased temperature levels. Front Microbiol 7:434 27065988 10.3389/fmicb.2016.00434 PMC4814934

[92] Paix B, Potin P, Schires G, le Poupon C, Misson B, Leblanc C et al (2021) Synergistic effects of temperature and light affect the relationship between *Taonia atomaria* and its epibacterial community: a controlled conditions study. Environ Microbiol 23:6777–6797 34490980 10.1111/1462-2920.15758

[93] Staehr PA, Wernberg T (2009) Physiological responses of *Ecklonia radiata* (laminariales) to a latitudinal gradient in ocean temperature. J Phycol 45(1):91–99 27033648 10.1111/j.1529-8817.2008.00635.x

[94] Wernberg T, Thomsen MS, Tuya F, Kendrick GA, Staehr PA, Toohey BD (2010) Decreasing resilience of kelp beds along a latitudinal temperature gradient: potential implications for a warmer future. Ecol Lett 13(6):685–694 20412279 10.1111/j.1461-0248.2010.01466.x

[95] Becheler R, Haverbeck D, Clerc C, Montecinos G, Valero M, Mansilla A et al (2022) Variation in thermal tolerance of the giant kelp’s gametophytes: suitability of habitat, population quality or local adaptation? Front Mar Sci 9:802535

[96] Mancuso FP, Morrissey KL, de Clerck O, Airoldi L (2023) Warming and nutrient enrichment can trigger seaweed loss by dysregulation of the microbiome structure and predicted function. Sci Total Environ 879:162919 36958561 10.1016/j.scitotenv.2023.162919

[97] Marzinelli EM, Campbell AH, Zozaya Valdes E, Vergés A, Nielsen S, Wernberg T et al (2015) Continental-scale variation in seaweed host-associated bacterial communities is a function of host condition, not geography. Environ microbiol 17(10):4078–4088 26148974 10.1111/1462-2920.12972

[98] Saha M, Rempt M, Stratil SB, Wahl M, Pohnert G, Weinberger F (2014) Defence chemistry modulation by light and temperature shifts and the resulting effects on associated epibacteria of *Fucus vesiculosus*. PLoS ONE 9:e105333 25360717 10.1371/journal.pone.0105333 PMC4215838

[99] Zhang X, Xi T, Wang Y, Fan X, Xu D, Zhang P et al (2024) Chemical interactions between kelp *Macrocystis pyrifera* and symbiotic bacteria under elevated CO_2_ condition. Mar Life Sci Technol 6:700–712 39620087 10.1007/s42995-024-00259-5 PMC11602886

[100] Cavalcanti GS, Shukla P, Morris M, Ribeiro B, Foley M, Doane MP et al (2018) *Rhodolith* holobionts in a changing ocean: host-microbes interactions mediate coralline algae resilience under ocean acidification. BMC Genomics 19:1–13 30249182 10.1186/s12864-018-5064-4 PMC6154897

[101] Van der Loos LM, Steinhagen S, Stock W, Weinberger F, D’hondt S, Willems A, de Clerck O (2025) Low functional change despite high taxonomic turnover characterizes the *Ulva* microbiome across a 2000-km salinity gradient. Sci Adv 11:eadr6070 39823339 10.1126/sciadv.adr6070 PMC11740975

[102] Dittami SM, Duboscq-Bidot L, Perennou M, Gobet A, Corre E, Boyen C et al (2016) Host–microbe interactions as a driver of acclimation to salinity gradients in brown algal cultures. ISME J 10(1):51–63 26114888 10.1038/ismej.2015.104 PMC4681850

[103] Morrissey KL, Iveša L, Delva S, D’hondt S, Willems A, De Clerck O (2021) Impacts of environmental stress on resistance and resilience of algal-associated bacterial communities. Ecol Evol 11:15004–15019 34765156 10.1002/ece3.8184 PMC8571626

[104] Pei P, Aslam M, Du H, Liang H, Wang H, Liu X et al (2021) Environmental factors shape the epiphytic bacterial communities of *Gracilariopsis lemaneiformis*. Sci Rep 11:8671 33883606 10.1038/s41598-021-87977-3 PMC8060329

[105] Michotey V, Blanfuné A, Chevalier C, Garel M, Diaz F, Berline L et al (2020) In situ observations and modelling revealed environmental factors favouring occurrence of *Vibrio* in microbiome of the pelagic *Sargassum* responsible for strandings. Sci Total Environ 748:141216 32798861 10.1016/j.scitotenv.2020.141216

[106] Florez JZ, Camus C, Hengst MB, Buschmann AH (2021) A mesocosm study on bacteria-kelp interactions: importance of nitrogen availability and kelp genetics. J Phycol 57:1777–1791 34570392 10.1111/jpy.13213

[107] James AK, English CJ, Nidzieko NJ, Carlson CA, Wilbanks EG (2020) Giant kelp microbiome altered in the presence of epiphytes. Limnol Oceanogr Lett 5:354–362

[108] Lemay MA, Martone PT, Keeling PJ, Burt JM, Krumhansl KA, Sanders RD et al (2018) Sympatric kelp species share a large portion of their surface bacterial communities. Environ Microbiol 20(2):658–670 29124859 10.1111/1462-2920.13993

[109] Davis KM, Mazel F, Parfrey LW (2021) The microbiota of intertidal macroalgae *Fucus distichus* is site-specific and resistant to change following transplant. Environ Microbiol 23:2617–2631 33817918 10.1111/1462-2920.15496

[110] Roth-Schulze AJ, Pintado J, Zozaya-Valdés E, Cremades J, Ruiz P, Kjelleberg S et al (2018) Functional biogeography and host specificity of bacterial communities associated with the marine green alga *Ulva* spp. Mol Ecol 27:1952–1965 29420863 10.1111/mec.14529

[111] Capistrant-Fossa KA, Morrison HG, Engelen AH, Quigley CTC, Morozov A, Serrão EA et al (2021) The microbiome of the habitat-forming brown alga *Fucus vesiculosus* (*Phaeophyceae*) has similar cross-Atlantic structure that reflects past and present drivers. J Phycol 57:1681–1698 34176151 10.1111/jpy.13194

[112] Molnar NB, Weigel BL, Fales RJ, Pfister CA (2025) Warming Seawater Temperature and Nutrient Depletion Alters Microbial Community Composition on a Foundational Canopy Kelp Species. Environ Microbiol 27(3):e70077 40075558 10.1111/1462-2920.70077 PMC11903912

[113] Zozaya-Valdés E, Roth-Schulze AJ, Egan S, Thomas T (2017) Microbial community function in the bleaching disease of the marine macroalgae *Delisea pulchra*. Environ Microbiol 19:3012–3024 28419766 10.1111/1462-2920.13758

[114] Campbell AH, Harder T, Nielsen S, Kjelleberg S, Steinberg PD (2011) Climate change and disease: bleaching of a chemically defended seaweed. Glob Chang Biol 17:2958–2970

[115] Van der Loos LM, Eriksson BK, Salles JF (2019) The macroalgal holobiont in a changing sea. Trends Microbiol 27(7):635–650 31056303 10.1016/j.tim.2019.03.002

[116] Martignoni MM, Kolodny O (2024) Microbiome transfer from native to invasive species may increase invasion risk. Proceedings B 291(2034):20241318 10.1098/rspb.2024.1318 PMC11537765 39500380

[117] Bonthond G, Neu A, Bayer T, Krueger-Hadfield SA, Künzel S, Weinberger F (2023) Non-native hosts of an invasive seaweed holobiont have more stable microbial communities compared to native hosts in response to thermal stress. Ecol Evol 13(1):e9753 36713485 10.1002/ece3.9753 PMC9873590

[118] Samo TJ, Rolison KA, Swink CJ, Kimbrel JA, Yilmaz S, Mayali X (2023) The algal microbiome protects *Desmodesmus intermedius* from high light and temperature stress. Algal Res 75:103245

[119] Castro LC, Vergés A, Straub SC, Campbell AH, Coleman MA, Wernberg T et al (2024) Effect of marine heatwaves and warming on kelp microbiota influence trophic interactions. Mol Ecol 33:e17267 38230446 10.1111/mec.17267

[120] Ghaderiardakani F, Quartino ML, Wichard T (2020) Microbiome-dependent adaptation of seaweeds under environmental stresses: a perspective. Front Mar Sci 7:575228

[121] Ghotbi M, Bonthond G, Ghotbi M, Künzel S, Needham DM, Weinberger F (2024) Greater host influence and promiscuity: How an invasive seaweed host has advantages over co-occurring natives. bioRxiv 2012–24. 10.1093/ismeco/ycaf120 PMC12449580 40980730

[122] Bonthond G, Bayer T, Krueger-Hadfield SA, Stärck N, Wang G, Nakaoka M et al (2021) The role of host promiscuity in the invasion process of a seaweed holobiont. ISME J 15:1668–1679 33479490 10.1038/s41396-020-00878-7 PMC8163768

[123] Dai C, Wang S (2022) The structure and function of the *Sargassum fusiforme* microbiome under different conditions. J Mar Sci Eng 10:1401

[124] Aires T, Serrão EA, Kendrick G, Duarte CM, Arnaud-Haond S (2013) Invasion is a community affair: clandestine followers in the bacterial community associated to green algae, Caulerpa racemosa, track the invasion source. PLoS ONE 8(7):e68429 23874625 10.1371/journal.pone.0068429 PMC3713043

[125] Wang H, Elyamine AM, Liu Y, Liu W, Chen Q, Xu Y et al (2022) Hyunsoonleella sp HU1–3 increased the biomass of Ulva fasciata. Front Microbiol 12:788709 35173690 10.3389/fmicb.2021.788709 PMC8841488

[126] Saha M, Weinberger F (2019) Microbial “gardening” by a seaweed holobiont: surface metabolites attract protective and deter pathogenic epibacterial settlement. J Ecol 107:2255–2265

[127] Nair S, Zhang Z, Wang X, Zhang B, Jiao N, Zhang Y (2024) Engineering microbiomes to enhance macroalgal health, biomass yield, and carbon sequestration. Green Carbon 2024.

[128] Yarbro J, Khorunzhy E, Boyle N (2024) The phycosphere and its role in algal biofuel production. Front Clim 6:1277475

[129] Saravanan P, Chatterjee A, Kiran KJ, Bhowmick GD, Sappati PK, Nagarajan V (2024) Exploring seaweed-associated marine microbes: growth impacts and enzymatic potential for sustainable resource utilization. Indian J Microbiol 64:593–602 39011007 10.1007/s12088-024-01205-w PMC11246340

[130] Schmidt R, Saha M (2021) Infochemicals in terrestrial plants and seaweed holobionts: current and future trends. New Phytol 229:1852–1860 32984975 10.1111/nph.16957

[131] Wood G, Steinberg PD, Campbell AH, Vergés A, Coleman MA, Marzinelli EM (2022) Host genetics, phenotype and geography structure the microbiome of a foundational seaweed. Mol Ecol 31:2189–2206 35104026 10.1111/mec.16378 PMC9540321

[132] Corinaldesi C, Bianchelli S, Candela M, Dell’Anno A, Gambi C, Rastelli E et al (2023) Microbiome-assisted restoration of degraded marine habitats: a new nature-based solution? Front Mar Sci 10:1227560

[133] Fujita H, Ushio M, Suzuki K, Abe MS, Yamamichi M, Iwayama K et al (2023) Alternative stable states, nonlinear behavior, and predictability of microbiome dynamics. Microbiome 11:63 36978146 10.1186/s40168-023-01474-5 PMC10052866

[134] Buschmann AH, Camus C, Infante J, Neori A, Israel Á, Hernández-González MC et al (2017) Seaweed production: overview of the global state of exploitation, farming and emerging research activity. Eur J Phycol 52(4):391–406

[135] Cai J, Lovatelli A, Aguilar-Manjarrez J, Cornish L, Dabbadie L, Desrochers A, et al. (2021) Seaweeds and microalgae: an overview for unlocking their potential in global aquaculture development. FAO Fisheries and Aquaculture Circular 1229.

[136] Ward GM, Faisan JP Jr, Cottier-Cook EJ, Gachon C, Hurtado AQ, Lim PE et al (2020) A review of reported seaweed diseases and pests in aquaculture in Asia. J World Aquac Soc 51(4):815–828

[137] Doering T, Maire J, van Oppen MJH, Blackall LL (2023) Advancing coral microbiome manipulation to build long-term climate resilience. Microbiol Aust 44:36–40

